# Give me a pain that I am used to: distinct habituation patterns to painful and non-painful stimulation

**DOI:** 10.1038/s41598-021-01881-4

**Published:** 2021-11-25

**Authors:** Katharina Paul, Martin Tik, Andreas Hahn, Ronald Sladky, Nicole Geissberger, Eva-Maria Wirth, Georg S. Kranz, Daniela M. Pfabigan, Christoph Kraus, Rupert Lanzenberger, Claus Lamm, Christian Windischberger

**Affiliations:** 1 MR Center of Excellence, Center for Medical Physics and Biomedical Engineering, Medical University of Vienna, Waehringer Guertel 18-20, 1090 Vienna, Austria; 2 Social, Cognitive and Affective Neuroscience Unit, Department of Cognition, Emotion, and Methods in Psychology, Faculty of Psychology, University of Vienna, Vienna, Austria; 3 Department of Psychiatry and Behavioral Sciences, Stanford University, Stanford CA, USA; 4 Department of Psychiatry and Psychotherapy, Medical University of Vienna, Vienna, Austria; 5 Department of Rehabilitation Sciences, The Hong Kong Polytechnic University, Hong Kong, China; 6 Department of Behavioural Medicine, Faculty of Medicine, University of Oslo, Oslo, Norway

**Keywords:** Perception, Insula, Limbic system, Sensory processing

## Abstract

Pain habituation is associated with a decrease of activation in brain areas related to pain perception. However, little is known about the specificity of these decreases to pain, as habituation has also been described for other responses like spinal reflexes and other sensory responses. Thus, it might be hypothesized that previously reported reductions in activation are not specifically related to pain habituation. For this reason, we performed a 3 T fMRI study using either painful or non-painful electrical stimulation via an electrode attached to the back of the left hand. Contrasting painful vs. non-painful stimulation revealed significant activation clusters in regions well-known to be related to pain processing, such as bilateral anterior and posterior insula, primary/secondary sensory cortices (S1/S2) and anterior midcingulate cortex (aMCC). Importantly, our results show distinct habituation patterns for painful (in aMCC) and non-painful (contralateral claustrum) stimulation, while similar habituation for both types of stimulation was identified in bilateral inferior frontal gyrus (IFG) and contralateral S2. Our findings thus distinguish a general habituation in somatosensory processing (S2) and reduced attention (IFG) from specific pain and non-pain related habituation effects where pain-specific habituation effects within the aMCC highlight a change in affective pain perception.

## Introduction

Pain perception is crucial for survival as the basis for preserving physical integrity through active pain avoidance. However, adapting to persistent and non-avoidable pain is an important mechanism, as it allows preserving physical, emotional, and cognitive resources. The dramatic impact of a failure within this process becomes apparent in pathological chronic pain which leads to serious impairments in daily quality of life^1^ and even increased mortality related to pathological stress levels and abnormal endocrine stress responses^2^. In particular, syndromes associated with chronic pain seem to be characterized by defective habituation to painful events^3–5^, emphasizing the clinical relevance of insights into central habituation processes.

Pain habituation is commonly referred to as the adjustment to continuous or repetitive pain, resulting in a decrease in perceived pain intensity and pain-related responses. Habituation has been described repeatedly for subjective pain reports^6–11^ and has been shown to go along with changes in electrodermal activity, reflecting autonomic responses to pain^12,13^. In addition, brain activity as assessed using functional magnetic resonance imaging (fMRI) has also been shown to habituate during repeated painful stimulation, predominately in cingulate, insular and somatosensory cortices^10,12,14–17^, even when repetition was delayed by several days^18^.

Although brain response to painful events and its modulation by cognitive and emotional conditions has already been studied extensively^19–22^, the detailed mechanism underlying pain habituation is still unclear. In general, painful sensations by nociceptors are transferred via the dorsal root or trigeminal pathway and lateral spinothalamic tract to the cerebrum, where the insula, the pregenual anterior cingulate cortex (pACC), anterior midcingulate cortex (aMCC), thalamus, prefrontal and somatosensory cortices are involved in pain perception. These brain regions are referred to as the central components of the pain matrix^21,23,24^. Inhibition of this pathway via descending endogenous antinociceptive mechanisms is mediated by opioidergic mechanisms in the periaqueductal grey projecting to the medulla and the noradrenergic locus coeruleus^25,26^. With respect to pain habituation, wide parts of the cingulate cortex seem to play a crucial role in endogenous pain control^10,12,18^, which is supported by linkages to pain modulatory mechanisms like emotional state and placebo analgesia^27–30^.

Although habituation effects to painful stimuli have been studied and described repeatedly at various levels (subjective reports, autonomic responses, fMRI), little is known about the specificity of these effects, such as whether or not carefully matched non-painful stimuli show habituation effects similar to those associated with painful stimuli. In fact, it is likely that habituation to somatosensory input is not specific to painful stimulation^31,32^, since it has been described for other body responses as well, like spinal reflexes^33,34^ and auditory and visual evoked potentials^35,36^. This in turn raises the question whether the previously reported activation decay reflects a unique brain response to repetitive nociceptive sensations, or a more general adjustment to repeated (somato-)sensory input.

Previous studies only contrasted the response to nociceptive stimulation over time and could not show a specificity of habituation processes to pain^10,12,14,18^. As a result, knowledge on the specificity of habituation patterns to either nociceptive or pure somatosensory perception is extremely limited. Thus, we performed an fMRI experiment to study short-term habituation to nociceptive as well as to non-nociceptive stimulation. Using a well-established electrical stimulation protocol, we aimed to provide insights in the functional specificity of habituation within the pain matrix, i.e. a decrease in brain activity assessed with the BOLD signal in response to a repetitive painful or non-painful electric stimulation, to distinguish regions with pain-specific from those with non-specific habituation patterns.

## Methods

### Participants

Twenty-three subjects (11 females, mean age = 24.48, SD = 4.42) participated in this study. All subjects were right-handed as confirmed with the Edinburgh Handedness Inventory^37^, had normal or corrected-to-normal vision, reported no history or acute neurological or psychiatric disorders (assessed with the Structural Clinical Interview for DSM-IV, SCID), past or present substance abuse, or prolonged use of psychopharmacological medication (including pain killers) within the last 3 months. They gave written informed consent and received reimbursement for participation. The study was performed in line with the latest revision of the Declaration of Helsinki, and approved by the ethics committee of the Medical University of Vienna.

### Pain task

Within the paradigm, painful and non-painful stimuli were applied with a custom-made electrode with 7 mm diameter and a platinum pin (WASP electrode, Specialty Developments) using an isolated bipolar constant current stimulator (DS5, Digitimer Ltd, Hertfordshire, UK) placed at the dorsum of the left hand, between thumb and index finger^38–40^, a setting that has been successfully used in previous studies^41–43^. We determined electrical currents individually to account for inter-subject variability in pain thresholds by increasing the intensity stepwise and using a 10-point Likert scale, anchored with “detectable sensation (1)” to “worst imaginable pain (10)”. Non-painful stimuli were ensured to be perceptible but not uncomfortable (rated as 1). Painful stimuli were calibrated to be painful but still tolerable (rated with 6) to ensure that the participant can withstand the stimulation for the whole experiment. While this procedure led to varying thresholds (ranging from 0.01 to 0.4 mA (M = 0.10) for non-painful and 0.1 to 1.9 mA (M = 0.50) for painful stimulation), it ensured that the stimuli were perceived as intended and similarly across all subjects. As the current experiment did not include any ratings on experienced pain intensity, we run an additional experiment to collect information on possible habituation effects in subjective pain experience, see Supplementary Analyses.

**Figure 1:**
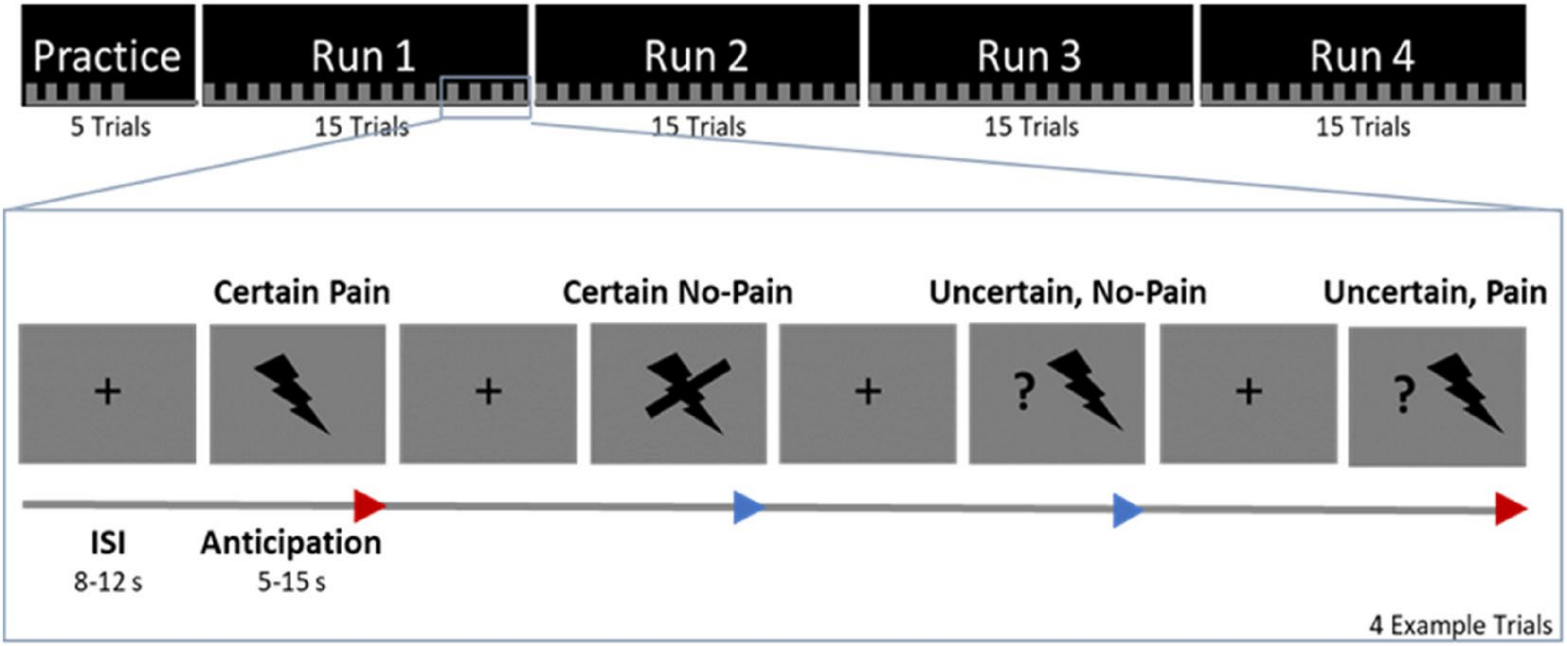
Overview of the experimental paradigm. After calibration and a short practice, participants did four blocks of the experiment with 15 trials each. Overview of the experimental paradigm. Painful and non-painful stimuli were administered in randomized order. Cues indicating the type of trial were presented 5–15 s prior the stimulus and persisted during the actual transcutaneous electrical stimulation. A thunderbolt cued for a painful stimulation, a crossed-out thunderbolt for a non-painful trial. The thunderbolt with question mark indicated an uncertain trial (50% painful/non-painful) and therefore provided no information about the upcoming stimulation.

During the experiment, participants were told to prepare for the upcoming trial and concentrate on the painful or non-painful sensation. Three trial types were indicated by one of three different visual cues (black symbols on gray background), which preceded the electric stimulation (see Fig. 1). Painful stimuli were indicated by a thunderbolt, a crossed-out thunderbolt indicated a non-painful stimulus. A thunderbolt with a question mark indicated that the next stimulus could be either painful or non-painful (50% chance). All participants were informed about the meaning of each anticipation cue. The cue was shown for 5–15 s (normally distributed, on average 10.43 s (SD = 2.71) and was further presented during the delivery of the electric stimulus (500 ms). These preceding visual cues made the participants attentive to the task and allowed participants to prepare for the upcoming stimulation. A crosshair was presented between each stimulation trial for 8 to 12 s. Trial order was randomized within four separate runs, each containing 15 stimuli (5 painful, 5 non-painful, 5 unsure) and lasting for about six minutes. Before the actual experiment, participants completed five training trials outside the scanner. Both stimulus presentation and stimulus delivery were controlled with Cogent (2000 v1.32), implemented in MATLAB (The Mathworks Inc., Sherborn, MA, USA).

### fMRI acquisition and statistical analysis

MR images were acquired on a 3 Tesla TIM Trio MR scanner (Siemens Medical, Erlangen, Germany) using the manufacturer’s 32-channel head coil. Functional whole-brain volumes were obtained using a single-shot gradient-recalled EPI-sequence. Scanning parameters were TE/TR = 38/1800 ms with 23 slices and a voxel size of 1.48 × 1.48 × 3 mm^3^ plus 1.8 mm slice gap (acquisition matrix = 128 × 128).

Data preprocessing followed an in-house made routine including different software packages, to choose the most capable procedure for each step. During preprocessing, the acquired data was despiked using AFNI (https://afni.nimh.nih.gov/), slice-timing corrected using FSL5 to the middle slice^44^, bias-field and distortion corrected using ANTs (http://stnava.github.io/ANTs/), realigned using FSL5 (http://fsl.fmrib.ox.ac.uk/fsl/fslwiki/), normalized to standard symmetric MNI space using ANTs in combination with a custom in-house built scanner-specific EPI-template and then smoothed with a 6 mm FWHM Gaussian kernel using FSL5. Finally, for each participant a GLM was defined using SPM12 revision 6225 (http://www.fil.ion.ucl.ac.uk/spm/). Regression at each voxel was estimated using generalized least squares with a global approximate AR (1) autocorrelation model and drift fit with Discrete Cosine Transform basis (128 s).

Nineteen regressors were defined for each of the four runs, featuring the stimulation periods (painful as expected, non-painful as expected, uncertain painful, uncertain non-painful) and the three anticipation phases (painful, non-painful, uncertain) with varying time-windows, convolved with the canonical hemodynamic response function as implemented in SPM. Additional nuisance regressors included realignment parameters and potentially confounding signals from white matter and ventricles for a detailed description see^45^.

For the second-level analyses, we restricted the design to two trial types: expected painful stimulation and expected non-painful stimulation. Uncertain conditions were omitted to exclude emotional confounds (e.g., elicited by surprise). These were in the scope of another analysis^46^. In order to reveal pain-related neural effects we specified a flexible factorial model including the above contrasts (for each of the four runs painful as expected and non-painful as expected, ending up with eight regressors) of the first-level model and examined differences between expected painful and expected non-painful stimulation. This analysis was performed to reveal the pain matrix^21,23^ and to confirm that our stimulation approach was successful.

Group statistics were calculated using second-level random effects analyses in SPM12. Results are presented and interpreted at a cluster-level corrected threshold of p < 0.05 (initial uncorrected threshold p < 0.001) using cluster-level correction based on the random Gaussian field approach as implemented in SPM 12, if not specified differently.

In order to test for habituation effects, we additionally contrasted activation from the first (run #1) and the last run (run #4) of each participant using paired t-tests, separately for painful and non-painful stimulation. In a second step, MARSBAR^47^ was used to create spherical ROIs (r = 4 mm) around the peak voxels in these two habituation contrasts (first run compared to last run, separately for pain and non-painful stimulation, see Fig. 3). Mean percent signal changes based on the individual beta values extracted from the single-subject analyses for each ROI, condition (painful and non-painful) and task block (first, second, third, fourth run) were extracted using MARSBAR. This approach did not assume linear habituation changes and, thus, allowed for an unbiased assessment of habituation-related effects over time, i.e., across all four runs, and between the painful and non-painful stimulation. This way we chose an unbiased but very sensitive approach independently for each condition to reveal any decrease in BOLD response activation over time. In an exploratory approach, we also focused on the habituation during the anticipation of (non)painful stimulation, as well as habituation within runs (as opposed to across runs as presented here), see Supplementary Analyses.

In addition, linear regression analysis, as implemented in MATLAB, was applied on the individual task-related signal changes followed by a one-sample t–test of the estimated regression parameters to test for significant linear habituation effects for each ROI and condition (significance level p < 0.01).

In a supplementary analysis, an exponential function was used to fit the task-related signal changes and the interaction effect of run and condition was evaluated within these ROIs (see Supplementary section).

### Subjective pain ratings

As the experiment reported above did not include any subjective ratings of perceived pain intensity, we could not determine if habituation occurred at experience levels similarly to the reported habituation of brain activity. While habituation in subjective pain ratings has been reported widely^15,18,48^, we could not find any habituation effects in similar studies with varying set-ups^43^. In order to explore possible habituation of subjective levels with the current experiment, we collected data in 10 additional subjects. The experimental procedure was identical to the one reported in the main experiment but participants were instructed to rate the electric stimulation they just received. For that, they used a four-point scale, anchored with “detectable sensation (1)” to “worst imaginable pain (4)” and reported their answer via button-press.

To estimate the experienced habituation over blocks, we followed a similar approach as in the main analysis: for each subject, we applied linear regression analysis as implemented in R on the individual ratings for each condition followed by a one-sample t-test of the estimated regression parameters (significance level p < 0.01).

## Results

### Task related effects: the pain matrix

**Figure 2:**
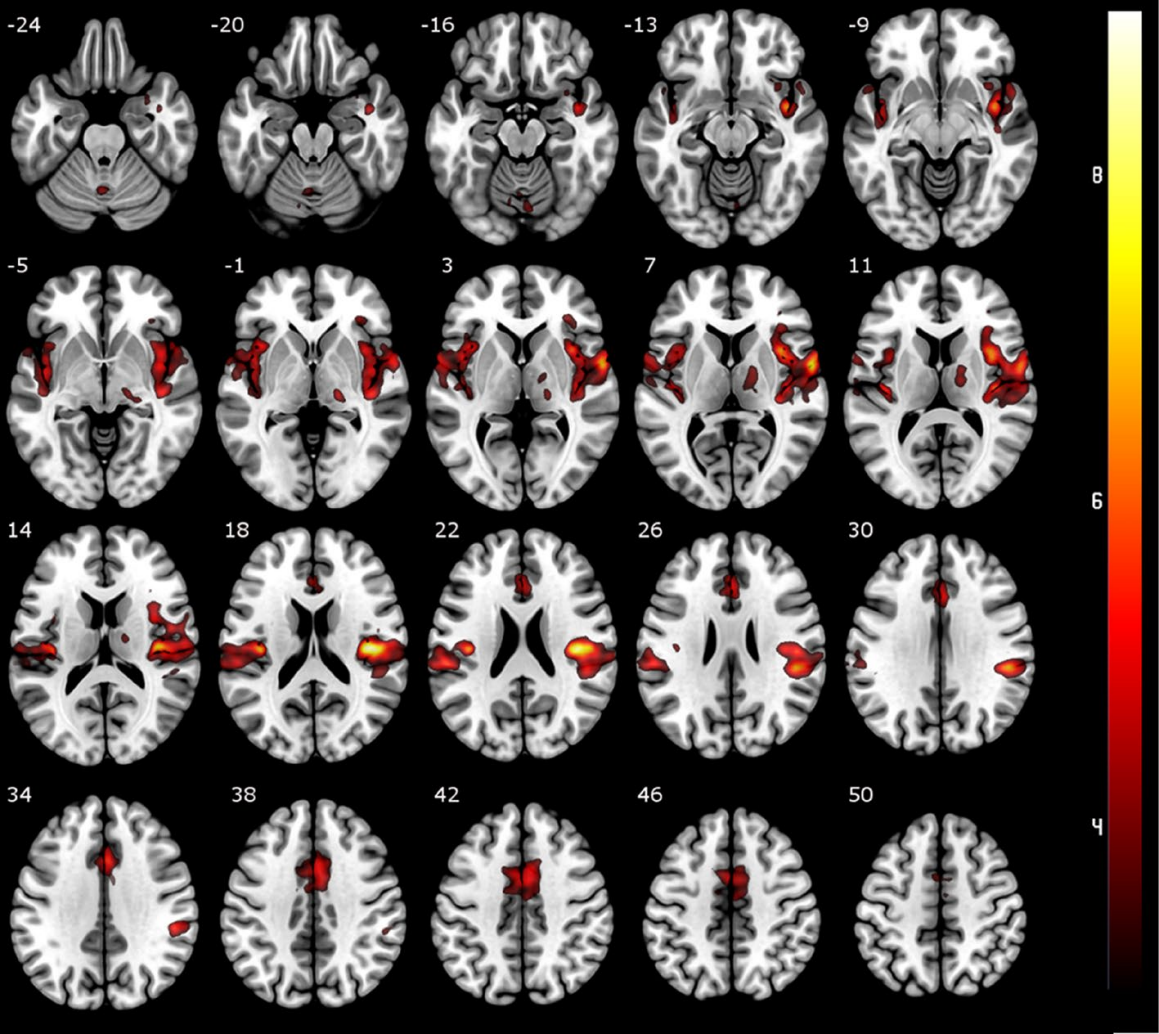
Results of the contrast painful vs. non-painful stimulation (all runs included). Significant activation differences were observed in areas typically involved in pain processing such as the insula, midcingulate and somatosensory cortices. Results are presented at a cluster-level family-wise error (FWE) corrected threshold of p < 0.05.

Although the concept of a pain-specific matrix has been contested^49,50^, regions related to processing of painful stimuli were determined by contrasting expected painful vs. expected non-painful stimulation. Significant activation was identified in regions typically associated with pain processing, including bilateral anterior and posterior insula, extending to the primary and secondary sensory cortices (S1/S2), aMCC, parts of the right thalamus and the left cerebellum (p < 0.05 FWE cluster-level corrected, see Table 1 and Fig. 2). The reverse contrast (expected non-painful > expected painful stimulation) showed no statistically significant activation.

**Table 1:** Significant brain activation clusters for contrast painful vs. non-painful stimulation with cluster size (k), t-value and MNI coordinates.

Activation clusters for painful vs. non-painful stimulation					
Region	k (vx)	*t*	MNI coordinates (mm)		
			x	y	z
Insula right	10,399	9.06	40	− 19	20
Insula left	5594	7.11	− 39	− 19	20
aMCC	2944	5.76	3	14	35
thalamus	571	4.65	18	− 22	− 1
Pyramis	574	4.43	− 6	− 72	− 31

### Habituation-related effects

**Figure 3:**
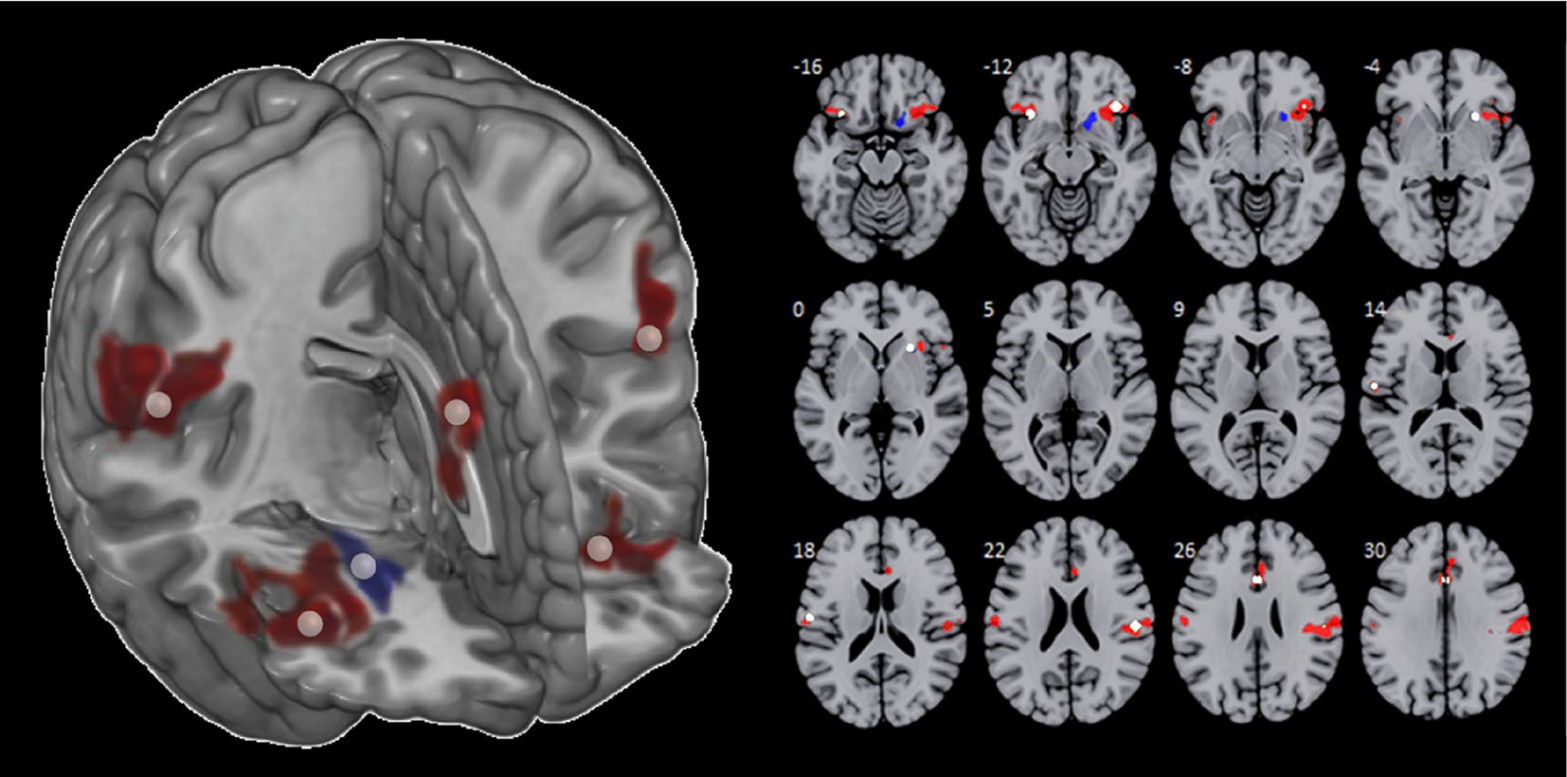
Habituation effects for painful and non-painful stimulation. Clusters showing habituation-related effects (Run 1 > Run 4) separately for painful (red) and non-painful stimulation (blue). ROI definition based on peak voxels as center for 4 mm spherical ROIs (white spheres) that were used in subsequent analyses.

Contrasting activation to painful stimuli in the first vs. the last run using t-tests revealed habituation effects in bilateral inferior frontal gyrus (IFG) including anterior parts of the insula, bilateral S1/S2, and the aMCC (p < 0.05 FWE cluster-level corrected). For non-painful stimuli, habituation effects were only found in the right claustrum. Figure 3 shows the corresponding activation maps, and Table 2 lists the individual cluster details.

**Table 2:** Pain habituation.

Run 1 > run 4							Significant linear decrease	
Contrast	Region	k	*t*	MNI coordinates (mm)				
				x	y	z	Pain	No pain
Pain	IFG right	1125	6.96	38	26	− 12	*	*
	S2 right	1138	6.8	54	− 20	22	*	*
	aMCC	552	6.27	0	18	28	*	
	S2 left	300	5.38	− 58	− 13	16	*	
	IFG left	369	5.26	− 32	20	− 14	*	*
No pain	claustrum right	356	5.57	24	18	− 2		*

Significant brain activation clusters for contrast first vs. fourth run, separately for painful and non-painful stimulation with cluster size (k), t-value and MNI coordinates. Only the highest peak is included in case of several confluent peaks.

**Figure 4:**
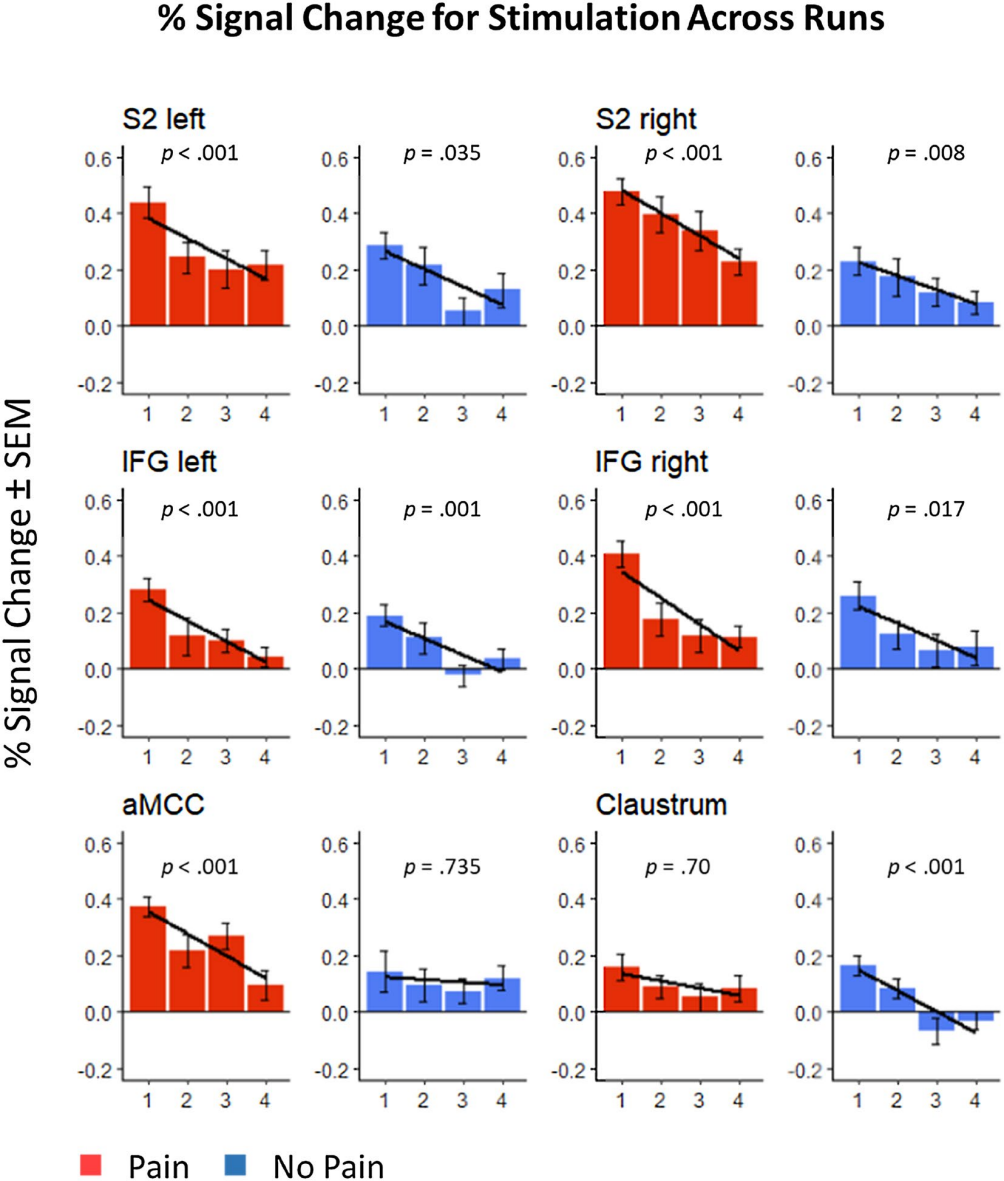
Contrast estimates of painful and non-painful stimulation over the four runs. This figure shows habituation effects within S2, IFG, aMCC and claustrum, comparing mean BOLD responses, separately for painful (red bars) and non-painful stimulation (blue bars). Black lines indicate a significant linear decrease over the four runs (p < 0.01). While there was a significant habituation to both kind of stimulations within bilateral IFG and right S2, habituation was found within the left S2 and the aMCC only for pain, while the claustrum showed habituation only to non-painful stimulation.

For these clusters, linear regression analysis as implemented in Matlab was performed to test for habituation over all four runs (see Fig. 4). All areas with significant differences between first and fourth run for painful or non-painful stimulation also showed a significant linear decrease in activation levels across runs. Interestingly some areas showed this linear decrease although they did not show a significant difference in the contrast between first and fourth run, indicating that more subtle habitation effects could be detected with these analyses. Thus, within bilateral IFG and right S2, activation was found to decrease linearly over runs for both painful and non-painful stimulation, whereas the left (contralateral) S2 and the aMCC showed habituation effects for painful stimulation only. Habituation effects restricted to non-painful stimulation were found within parts of the right claustrum extending to the putamen (all p < 0.001).

### Subjective pain ratings

**Figure 5:**
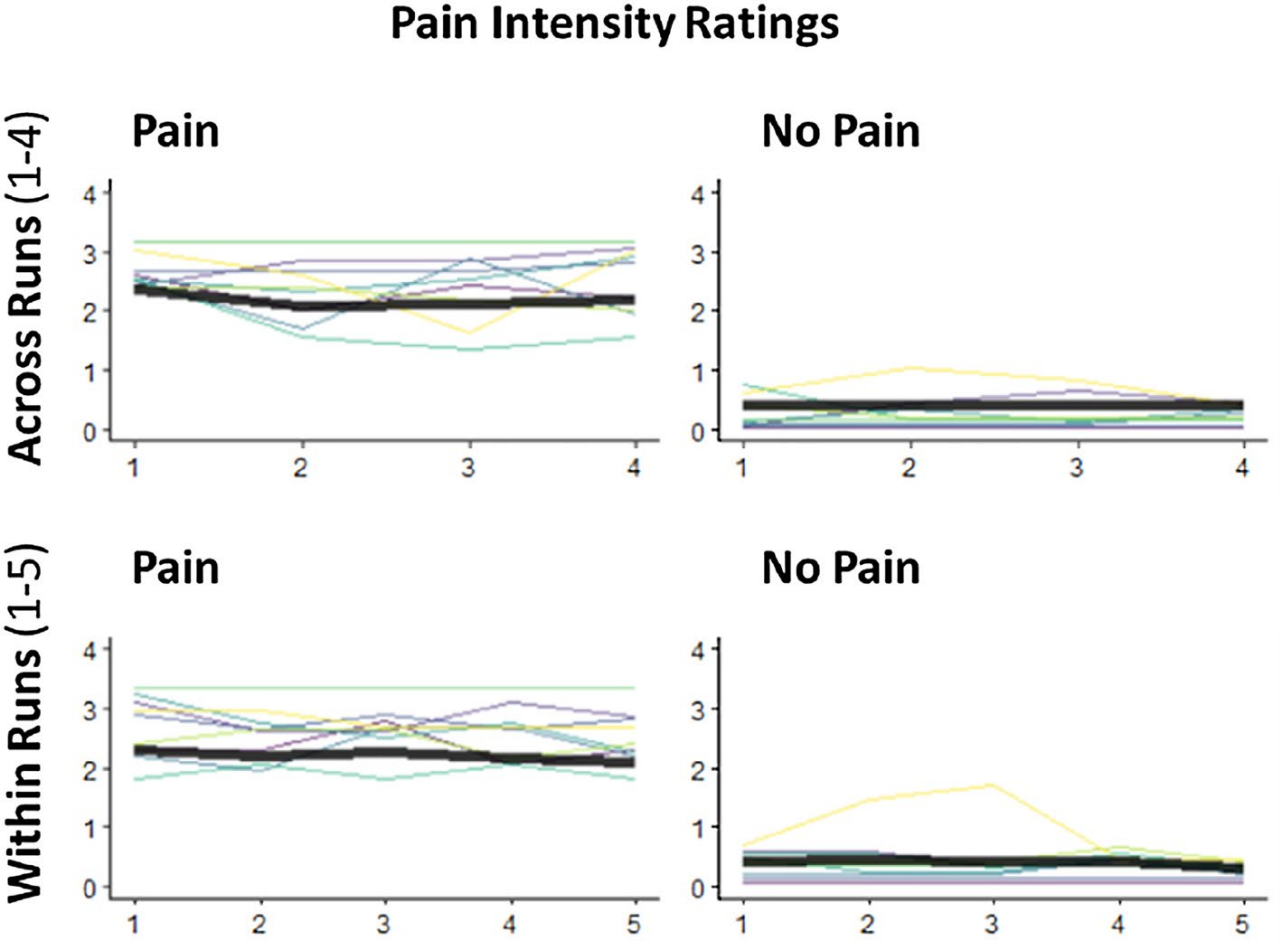
Subjective Ratings of Pain Intensity. Average ratings of experienced pain “1 = detectable sensation” to “4 = worst imaginable pain” are shown over a) runs (upper row) and within Runs (lower row), separately for painful and non-painful stimulation. Bold black lines correspond to mean rating across subjects; color lines correspond to individual ratings averaged across trials.

As seen in Fig. 5, participants’ ratings did not differ over time, as average ratings per block were rather stable across the four runs (see Supplementary Table 1). Together with the visualization of the individual ratings (see Fig. 5) as well as similar findings in our related studies^43^, this suggests that participants’ subjective ratings of pain intensity did not habituate over the experiment.

## Discussion

In this study, we assessed the specificity of short-term habituation effects of brain activity to individually determined painful and non-painful stimulation. As expected, a network of brain regions associated with pain processing (Insula, ACC, S1/S2, thalamus) showed increased activation during painful compared to non-painful electric stimulation^21,23^. Focusing on habituation effects throughout the experiment revealed a common decrease in stimulation-related activation changes within parts of this network (anterior insula, S2) and further inferior frontal regions, irrespective of the stimulation type (painful, non-painful). Habituation effects restricted to painful stimulation only occurred within the aMCC and the contralateral S2, while the BOLD signal within the claustrum decreased for non-painful stimuli only. These results highlight that habituation to painful and non-painful stimulation is underpinned by both general habituation to repeated somatosensory stimulation and specific changes in the motivational response to persistent nociceptive stimulation.

Habituation to painful stimulation within wide and extending parts of the ACC/MCC has been reported previously^12,14,16^. Here, we were able to classify these habituation processes within the aMCC as a specific characteristic of pain processing, as no habituation effects were observed in response to non-painful stimulation (as indicated by both, the fitting of a linear decrease and evaluating the interaction effect of condition and run). Among varying other functions^51^, the aMCC is an area commonly activated in pain studies, showing that activity within the aMCC does not code pain intensity but is more related to pain identification^52^. Since the aMCC is involved in many cognitive, affective and emotional processes, the actual role within pain processing is still object of intense debate, which has been set between both attentional and evaluative processes^21,53–55^. Importantly other areas of the “pain matrix”, including wide parts of the insula or the ACC, did not show a habituation effect. And although the concept of the “pain matrix” has been criticized in terms of its pain specificity^49,56^, it is still useful for the current manuscript, where habituation to painful is compared to the processing of a repeated tactile non-painful stimulation. The detected habituation effect does not seem to be related to a general decrease in pain-related activation, but to reflect a selective change, which we propose to be related to the emotional valuation and the motivational response to painful stimulation. One possibility to explain the pain-specific habituation within the aMCC refers to the four-region model of the cingulate cortex which suggests a role of the aMCC in fear avoidance behaviour^57,58^ based on strong connections from amygdala and the medial pain system (midline and intra laminar thalamic nuclei)^59^. Repeated exposure to frightening stimuli leads to a reduction in physiological responses and subjective fear experience, thus, overcoming avoidance behaviour has become one of the basic principles of desensitization therapy for anxiety disorders^60^. Keeping in mind that the aMCC is more linked to avoidance behaviour than to fear experience^57^, it seems obvious that the need for behavioural change decreases over time. This is further supported by the current task instruction to lie still during the scanning session and explicitly not to move the hand, as any reactive behaviour to pain perception had to be actively withheld by the participant.

On the other hand, the unspecific activation decrease found in the contralateral S2 for both stimulation types favours a classic sensory habituation effect. Activity of S2 is not specific to noxious stimuli but increases with perceived intensity and seems to be involved in general somatosensory integration^21^. The comparable pattern in response to painful and non-painful stimulation could indicate that the sensory features of painful and non-painful stimulation have changed over the experiment similarly. Since the habituation effect was not distinctive to non-painful stimulation, the habituation within S2 seems less related to differences in perceived pain intensity than to sensory habituation to repeated somatosensory stimulation. Complementary, we found a pain-specific decrease in BOLD signal at ipsilateral sides. Bilateral responses within S2 to painful stimulation are quite common^21^ and indicate the complex and widespread network of pain processing. Some^61^ found that bilateral activation gets stronger with higher stimulus intensity while others^62,63^ refined that this is only true for painful intensities. Likewise, our painful stimuli with their higher stimulation intensity compared to non-painful stimuli activated bilateral cortical areas at the beginning of the experiment. Our results further corroborate these findings indicating bilateral habituation effects preferentially upon painful stimulation while only a contralateral habituation effect was found for non-painful stimulation.

One further finding was the decrease in activation over time within bilateral parts of the inferior frontal gyrus (IFG) for both stimuli. This area is not part of the pain matrix in the first place, but has been reported in the context of discrimination of painful stimuli^64,65^. There, it showed higher activation when participants were instructed to differentiate between different stimulation intensities than when stimuli were not further rated, which is in line with the role of the IFG in attention towards (task) relevant events^66–68^, such as painful stimuli^69,70^. The decrease over time could therefore indicate a loss of attentive focus to stimulation to the same extent for painful and non-painful trials. Interestingly, other parts of the pain matrix (e.g., the contralateral insula) did not show any decrease over time and differentiated painful from non-painful stimulation over the entire experiment, which indicates that the brain consistently identified painful events, regardless how often the stimuli were presented, even though a potential lack of attention.

Another interesting result of this study is the habituation specific to no-painful stimuli occurring within parts of the contralateral claustrum. This finding seems very surprising at the first sight, as the claustrum is not part of the pain matrix and seems to be not involved in the distinction of painful vs. non-painful events. Nevertheless, a closer look at Supplementary Fig. 1 reveals that he claustrum was indeed activated by the stimuli, for both painful and non-painful stimulation however. While the function of the claustrum is still largely unknown, it is considered a synchronizing relay of cortical information or even a central network component for the emergence of consciousness^71,72^. Moreover, the claustrum shows strong reciprocal connections with large parts of the cortex, which makes it a suitable candidate region for multisensory integration^73^. Recently, it was proposed that the claustrum is part of a sensory association cortex-to-claustrum-to-cingulate pathway and could be involved in encoding salience of incoming stimuli^72^. In this view, the specific habituation for non-painful stimulation would be plausible, since a repeated non-harmful event will lose its saliency while the repeated painful stimulation will preserve it. Albeit our findings seem to support this view, the involvement of the claustrum in pain and/or salience processing is still unclear, quite speculative at present and needs further investigation in the future.

This experiment focussed on habituation of brain activity. Habituation in subjective pain ratings to repetitive thermal or electrical stimulation has been reported repeatedly^15,18,48^. Here, conclusions regarding the habituation of subjective experience could not be made directly, as the main experiment did not include behavioural ratings in order to avoid mapping neural processes related evaluation and rating. In an additional experiment with identical setting, we found no evidence of self-experienced habituation effects which is in line with other studies of our research group that were using a similar setup^43,74,75^. While this might highlight a relevant dissociation between subjective reports and brain activity, for similar arguments see^76,77^, future work should explicitly probe the link of habituation of brain activation with habituation of subjective experience. Relatedly, another limitation concerns the possible mechanisms of the reported habituation effects. While the current analysis focused on identifying distinctive habituation patterns for painful and non-painful stimulation, the cognitive or functional sources of these patterns remain an open question. In particular, since we used a previously recorded dataset, it could be possible that habituation is modulated by preparatory processes related to the anticipation of certain or uncertain events. A final limitation relates to possible differences in pain processing related to gender. While our sample is too small to make statistical comparisons between gender, women have been reported to be more sensitive to pain at neural levels and in their subjective reports^78,79^. However, the current design mitigates gender differences in different pain levels through the individually adjusted stimulation intensities. Nevertheless, it is possible that women and men might show different neural habituation patterns and more research is needed to shed light on these aspects.

In conclusion, we suggest re-evaluating the interpretation of previous findings that habituation effects in the somatosensory cortex are being specifically related to pain processing. Rather, we propose that these effects results from general habituation to repeated somatosensory stimulation. In contrast, areas such as the aMCC show genuinely pain-specific habituation effects, which we interpret as being related to a decrease in the affective-motivational response to persistent nociceptive stimulation.

## Supplementary Information

Supplementary Information.
