# Supplementary material for: Give me a pain that I am used to: distinct habituation patterns to painful and non-painful stimulation
Source: Sci Rep. 2021 Nov 25;11:22929. doi: 10.1038/s41598-021-01881-4 (PMC8617189; doi:10.1038/s41598-021-01881-4)
Supplement: Supplementary file 1 — Supplementary Information. [file 41598_2021_1881_MOESM1_ESM.docx]

**Supplementary to “Give me a pain that I am used to: Distinct habituation patterns to painful & non-painful stimulation”**

**Supplementary Table 1: Complete T-statistics of habituation across Runs**

| ROI | Condition | df |  | t | p |  | d | Mean [95% CI] |
| --- | --- | --- | --- | --- | --- | --- | --- | --- |
| Stimulation |  |  |  |  |  |  |  |  |
| S2_left | Pain | 22 |  | -4.19 | <.001 | * | 0.87 | -0.07 [-0.11;-0.04] |
| S2_right | Pain | 22 |  | -5.52 | <.001 | * | 1.15 | -0.08 [-0.11;-0.05] |
| IFG_left | Pain | 22 |  | -4.29 | <.001 | * | 0.89 | -0.07 [-0.11;-0.04] |
| IFG_right | Pain | 22 |  | -7.13 | <.001 | * | 1.49 | -0.09 [-0.12;-0.07] |
| ACC | Pain | 22 |  | -5.25 | <.001 | * | 1.1 | -0.08 [-0.11;-0.05] |
| Claustrum | Pain | 22 |  | -1.42 | 0.17 |  | 0.3 | -0.03 [-0.06;0.01] |
| S2_left | NoPain | 22 |  | -2.25 | 0.035 |  | 0.47 | -0.05 [-0.10;0.00] |
| S2_right | NoPain | 22 |  | -2.91 | 0.008 |  | 0.61 | -0.06 [-0.11;-0.02] |
| IFG_left | NoPain | 22 |  | -3.7 | 0.002 |  | 0.77 | -0.06 [-0.09;-0.03] |
| IFG_right | NoPain | 22 |  | -2.57 | 0.017 |  | 0.54 | -0.06 [-0.11;-0.01] |
| ACC | NoPain | 22 |  | -0.34 | 0.735 |  | 0.07 | -0.01 [-0.06;0.05] |
| Claustrum | NoPain | 22 |  | -5.62 | <.001 | * | 1.17 | -0.07 [-0.10;-0.05] |
|  |  |  |  |  |  |  |  |  |
| Anticipation |  |  |  |  |  |  |  |  |
| IFG_left | Pain | 22 |  | -4.13 | <.001 | * | 0.86 | -0.08 [-0.12;-0.04] |
| IFG_right | Pain | 22 |  | -5.15 | <.001 | * | 1.07 | -0.06 [-0.09;-0.04] |
| ACC | Pain | 22 |  | -4.21 | <.001 | * | 0.88 | -0.06 [-0.08;-0.03] |
| Thalamus | Pain | 22 |  | -5.23 | <.001 | * | 1.09 | -0.08 [-0.12;-0.05] |
| IFG_left | NoPain | 22 |  | -2.1 | 0.047 |  | 0.44 | -0.05 [-0.11;0.00] |
| IFG_right | NoPain | 22 |  | -3.27 | 0.004 |  | 0.68 | -0.05 [-0.08;-0.02] |
| ACC | NoPain | 22 |  | -1.2 | 0.243 |  | 0.25 | -0.03 [-0.07;0.02] |
| Thalamus | NoPain | 22 |  | -1.32 | 0.201 |  | 0.27 | -0.04 [-0.09;0.02] |
|  |  |  |  |  |  |  |  |  |
| Pain Intensity Rating | |  |  |  |  |  |  |  |
|  | NoPain | 9 |  | -0.17 | 0.87 |  | 0.05 | -0.005 [-0.07;0.05] |
|  | Pain | 9 |  | -0.93 | 0.38 |  | 0.29 | -0.04 [-0.14;0.06] |

**Supplementary Table 2: Complete T-statistics of habituation within Runs**

| ROI | Condition | df |  | t | p |  | d | Mean [95% CI] |
| --- | --- | --- | --- | --- | --- | --- | --- | --- |
| Stimulation |  |  |  |  |  |  |  |  |
| S2 left | Pain | 22 |  | -1.87 | 0.075 |  | 0.39 | -0.03 [-0.06;0.00] |
| S2 right | Pain | 22 |  | -1.95 | 0.064 |  | 0.41 | -0.03 [-0.06;0.00] |
| IFG left | Pain | 22 |  | -4 | <.001 | * | 0.83 | -0.06 [-0.09;-0.03] |
| IFG right | Pain | 22 |  | -5.14 | <.001 | * | 1.07 | -0.06 [-0.09;-0.04] |
| ACC | Pain | 22 |  | -4.21 | <.001 | * | 0.88 | -0.07 [-0.11;-0.04] |
| Claustrum | Pain | 22 |  | -1.54 | 0.138 |  | 0.32 | -0.02 [-0.05;0.01] |
| S2 left | NoPain | 22 |  | -1.8 | 0.085 |  | 0.38 | -0.07 [-0.15;0.01] |
| S2 right | NoPain | 22 |  | -1.92 | 0.067 |  | 0.4 | -0.06 [-0.13;0.00] |
| IFG left | NoPain | 22 |  | -2.83 | 0.010 |  | 0.59 | -0.12 [-0.21;-0.03] |
| IFG right | NoPain | 22 |  | -2.36 | 0.027 |  | 0.49 | -0.11 [-0.20;-0.01] |
| ACC | NoPain | 22 |  | -1.61 | 0.122 |  | 0.34 | -0.07 [-0.17;0.02] |
| Claustrum | NoPain | 22 |  | -1.25 | 0.223 |  | 0.26 | -0.06 [-0.16;0.04] |
|  |  |  |  |  |  |  |  |  |
| Anticipation |  |  |  |  |  |  |  |  |
| IFG left | Pain | 22 |  | -3.66 | 0.001 | * | 0.76 | -0.18 [-0.28;-0.08] |
| IFG right | Pain | 22 |  | -2.99 | 0.007 |  | 0.62 | -0.12 [-0.20;-0.04] |
| ACC | Pain | 22 |  | -2.35 | 0.028 |  | 0.49 | -0.08 [-0.16;-0.01] |
| Thalamus | Pain | 22 |  | -0.5 | 0.623 |  | 0.1 | -0.03 [-0.15;0.09] |
| IFG left | NoPain | 22 |  | -1.14 | 0.266 |  | 0.24 | -0.05 [-0.15;0.04] |
| IFG right | NoPain | 22 |  | -1.25 | 0.224 |  | 0.26 | -0.05 [-0.13;0.03] |
| ACC | NoPain | 22 |  | -1.29 | 0.209 |  | 0.27 | -0.05 [-0.14;0.03] |
| Thalamus | NoPain | 22 |  | 0.72 | 0.482 |  | 0.15 | 0.03 [-0.06;0.13] |
|  |  |  |  |  |  |  |  |  |
| Pain Intensity Rating | |  |  |  |  |  |  |  |
|  | NoPain | 9 |  | -1.27 | 0.235 |  | -0.02 | -0.06 [0.02;0.40] |
|  | Pain | 9 |  | -1.76 | 0.113 |  | -0.05 | -0.11 [0.01;0.56] |

**Within Run Habituation**

In order to explore habituation within runs (as opposed to across runs as described in the main section), we recalculated first level analysis to include separate regressors for each trial. Consequently, and similar to the main analysis, regressors were defined for each of the four runs, featuring one regressor for every event: 15 stimulation periods (painful as expected, non-painful as expected, uncertain painful, uncertain non-painful) and 15 anticipation phases (painful, non-painful, uncertain) with varying time-windows, convolved with the canonical hemodynamic response function as implemented in SPM. Additional nuisance regressors included realignment parameters and potentially confounding signals from white matter and ventricles (for a detailed description see Sladky et al., 2013). Similar to the main analysis, MARSBAR (Brett et al., 2002) was used to extract mean percent signal changes based on the individual beta values extracted from the single-subject analyses for each ROI, condition (5 painful and 5 non-painful trials) and task block (first, second, third, fourth run). In a next step, the mean percent signal changes were averaged across runs and linear regression analysis, as implemented in R, was applied on the individual task-related signal changes. Habituation was established by one-sample t–tests of the estimated regression parameters to test for significant linear habituation effects for each ROI and condition across subjects (significance level *p*<0.001).

As seen in Supplementary Figure 1 and Supplementary Table 1, habituation was only significantly established for painful stimulation within the ACC and bilateral IFG (*p* <.001). Neither for non-painful stimulation nor for other areas a significant linear decrease was found. Since these were post-hoc exploratory analyses, conclusions need to be drawn with caution. However, these results suggest that habituation of brain activity might occur already in relatively short time periods (less than a minute).


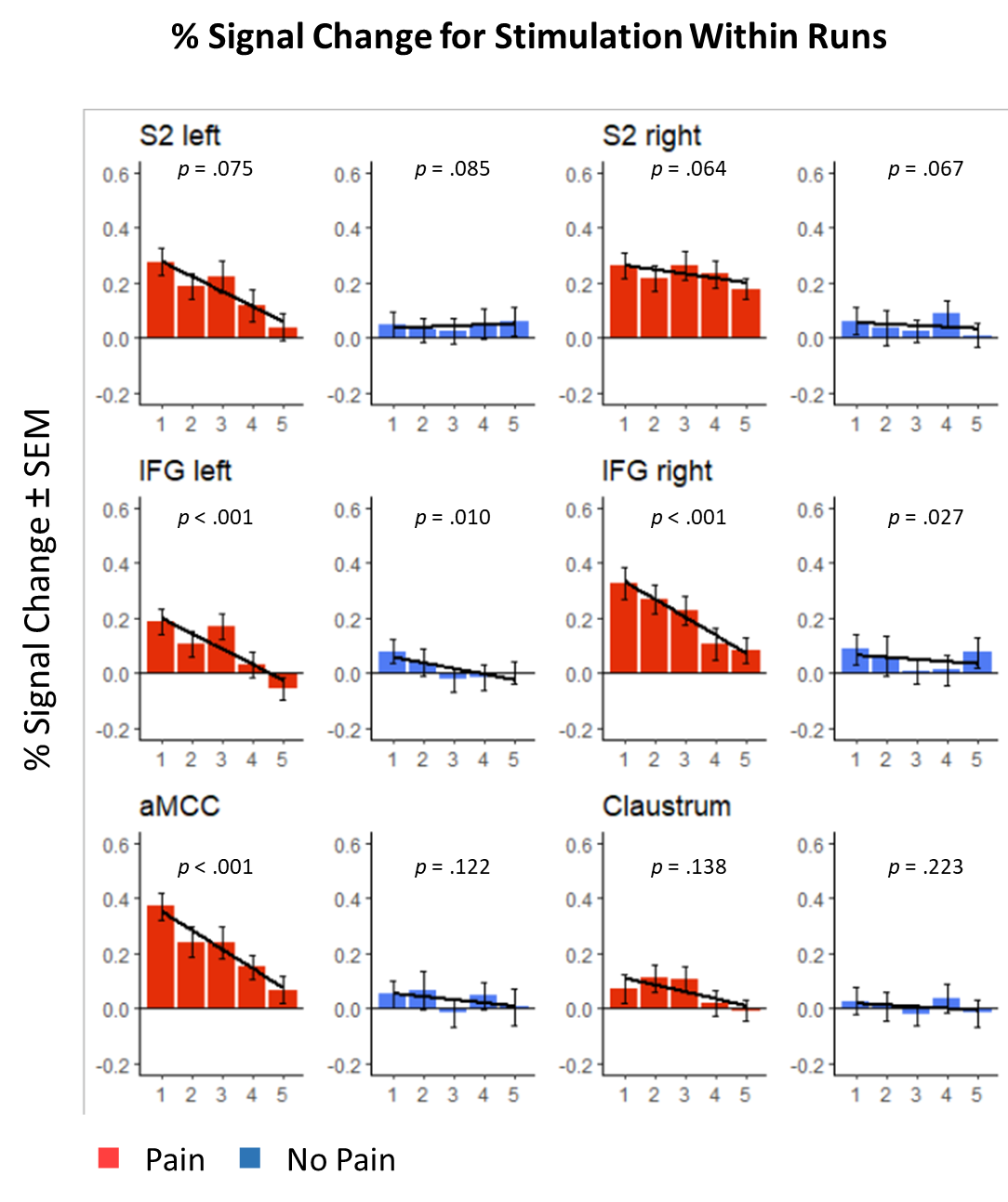


***Supplementary Figure 1. Contrast estimates of painful and non-painful stimulation over the five repetitions within a run.*** *This figure shows habituation effects within the bilateral IFGs and the ACC, but only for painful stimulation. Other regions, which show habituation over runs or also for non-painful stimulation, did not show any significant habituation within a run.*

**Habituation of Anticipation**

In order to explore habituation during the anticipation of painful and non-painful events, we redid all analysis reported also for the anticipation phase in identical ways juxtaposing the already reported approach. Since the anticipation phase was modeled at first-level analysis, we first contrasted the changes in anticipation between the first and the last run, separately for the anticipation of painful and non-painful stimulation. Only the contrast for Painful stimulation showed significant clusters (R1>R4) within the ACC, bilateral IFG and the thalamus, see Supplementary Table 3. Next, we created spherical 4mm ROIs around the peak activation of these clusters and extracted the mean percent signal change for each condition (or trial) using MARSBAR. Linear regressein analysis, as implemented in R, was applied on the individual task-related signal changes either across blocks (mean for each of the four runs, averaged across the five trials within one block) or within blocks (mean for each of the five trials, averaged across the four runs). Habituation was established by one-sample t–tests of the estimated regression parameters to test for significant linear habituation effects for each ROI and condition (significance level p<0.01). As seen in Supplementary Figure 2 and Supplementary Table 1, the pattern established from the main contrast was also found using this approach, but only for the anticipation of painful stimulation. When considering habituation within runs, only habituation for anticipation of painful stimulation was found in the left IFG. This indicates that selectively during the anticipation of painful stimuli, habituation during the anticipation phase occurred mainly over longer time intervals.

***Supplementary Table 3. Habituation of Anticipation.*** *Significant brain activation clusters for contrast first vs. fourth run, separately for anticipation for painful and non-painful stimulation with cluster size (k), t-value and MNI coordinates. Only the highest peak is included in case of several confluent peaks.*

| Run 1 > Run 4 | | | | | | |  |  | |  |  |
| --- | --- | --- | --- | --- | --- | --- | --- | --- | --- | --- | --- |
| Contrast | Region | k | *t* | MNI Coordinates (mm) | | |  | significant linear decrease | | |  |
|  |  |  |  | x | y | z |  | pain | no pain | | |
| pain | Thalamus | 368 | 5.47 | 8 | -18 | -6 |  | * | |  |  |
|  | IFG right | 917 | 5.46 | 42 | 20 | 10 |  | * | |  |  |
|  | IFG left | 320 | 5.14 | -38 | 25 | 4 |  | * | |  |  |
|  | aMCC | 1416 | 5.08 | 9 | 16 | 38 |  | * | |  |  |
| no pain | --- |  |  |  |  |  |  |  | |  |  |


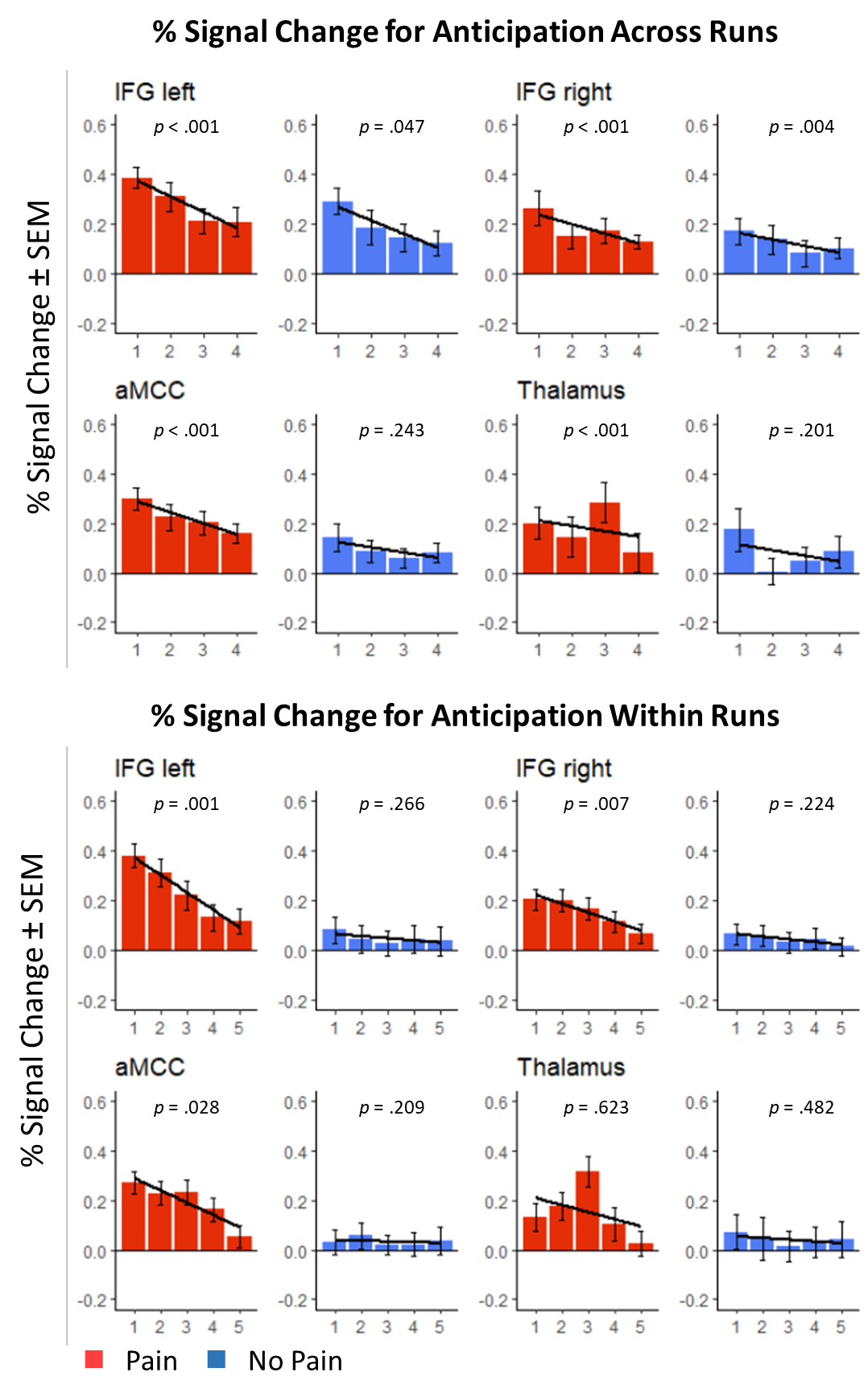


***Supplementary Figure 2. Contrast estimates of anticipation for painful and non-painful stimulation.*** *This figure shows habituation effects within the bilateral IFGs, the aMCC and the Thalamus, but only for anticipation of painful stimulation.*
